# Exploring Spatial Associations between On-Sale Alcohol Availability, Neighborhood Population Characteristics, and Violent Crime in a Geographically Isolated City

**DOI:** 10.1155/2013/356152

**Published:** 2013-11-27

**Authors:** Daikwon Han, Dennis M. Gorman

**Affiliations:** Department of Epidemiology & Biostatistics, School of Rural Public Health, Texas A&M University, College Station, TX 77843, USA

## Abstract

*Objectives*. Despite the increasing evidence of the associations between alcohol availability and violence, there are still inconsistent findings on the effects of on- and off-sale alcohol outlets on violent crime. The aim of this study was to examine spatial associations between on-sale alcohol availability, neighborhood characteristics, and violent crime in a geographically isolated city in Texas. *Methods*. Geographically weighted regression (GWR) and global regression models were employed to analyze the nature of the spatial relationship between violent crime, neighborhood sociocultural characteristics, and on-sale alcohol environment. *Results*. We found strong effects of neighborhood characteristics combined with on-sale alcohol availability on violence outcomes. Several neighborhood variables combined with alcohol availability explained about 63% of the variability in violence. An additional 7% was explained by the GWR model, while spatially nonstationary associations between violence and some predictor variables were observed. *Conclusions*. This study provided more credible evidence of the influence of on-sale alcohol outlets on violence in a unique setting. These findings have important policy implications in addressing the question of public health consequences of alcohol-related violence in local contexts.

## 1. Introduction

Two-decades of ecological research has demonstrated that alcohol-related violence is not simply an individual-level problem but rather must be understood within the community context, and that alcohol availability and the opportunity that this creates for drinking are an integral part of this problem [1]. Both cross-sectional and longitudinal studies have been used to assess the ecological relationship of various outlet density measures and place/population characteristics with violent crime across small geographic scales, including Census block groups [2], Census tracts [3], neighborhoods [4, 5], and zip codes [6, 7]. Although the specific relationship has varied by study setting, general findings show that neighborhoods with higher availability of alcohol are more likely to have higher rates of violent crime. There has also been some debate within the field of alcohol epidemiology as to whether alcohol outlet type, specifically on-sale versus off-sale premises, has differential effects on rates of violence. A recent study by Toomey and colleagues [5] found that the association with violence was stronger and more consistent in the case of on-sale than off-sale outlets although they noted that half of the previous studies that assessed on-sale outlets separately found no effect on violence. Moreover, there are ecological theories about drinking environments that focus on the role of on-sale outlets in attracting specific types of people into specific geographic locations and the types of interactions that occur within these microenvironments [8, 9].

Because there are still inconsistent findings of the effects of on-sale alcohol outlets on violent crime, this study was conducted to examine the nature of the relationship, with appropriate spatial methods, between on-sale alcohol availability, neighborhood population characteristics, and violent crime in a unique study setting. Specifically, the present study takes advantage of two features that distinguish it from previous research, and both of which arise from the nature of the study site, Lubbock, Texas. First, since alcohol was almost exclusively sold for on-premise consumption in Lubbock until September of 2009, this study site allows us to examine the effects of such premises on violence uncontaminated by the effect of off-sale premises. Second, Lubbock is geographically isolated from other large population centers and is surrounded by sparsely populated counties in which alcohol is not readily available. Thus, one might reasonably consider the sale and consumption of alcohol in Lubbock to be a closed system in which the alcohol-related problems that occur are likely to be a function of the alcohol sold within the city.

In addition, since spatial data are often characterized by two fundamental properties, spatial dependence and nonstationarity, statistical methods that are appropriate in spatial analyses were also employed to examine the nature of the spatial relationship between violent crime, neighborhood sociocultural characteristics, and on-sale alcohol environment. Specifically, Geographically Weighted Regression (GWR), combined with global regression models, was utilized in exploring local associations while taking into consideration spatial dependence and nonstationarity of the spatial data and identifying important covariates in the spatial associations between neighborhood population/place characteristics and violence.

## 2. Methods

### 2.1. Study Population and Data

As noted above, the city of Lubbock provides a fairly unique setting to investigate the relationship between on-sale alcohol availability and violence, because of its geographically isolated location and (prior to August, 2009) its retail alcohol market being comprised almost exclusively of on-premise outlets (only a handful of off-sale outlets existed in an area south of the city known as “The Strip”). The estimated population of the city for 2009 was 226,000, making it the 11th largest city in the state of Texas. The city is also home to Texas Technical University which had a student population of 30,049 in the fall semester of 2009. The closest population center to the north of Lubbock is Amarillo, which is 124 miles away and about a two-hour journey by car. The nearest cities after this (Oklahoma city, Fort Worth, Albuquerque, and Las Cruces) are each 300 to 400 miles away. In addition, the eight counties that surround Lubbock have low population density and three are totally “dry” (i.e., the sale of alcohol beverage is illegal). None of the remaining five is totally “wet”: about half of the precincts in four of these countries are dry, and the one county that has no dry precincts allows only off-premise sales of beer and wine.

As part of a larger study designed to examine the effects of the introduction of off-sale licenses in the city on September 23, 2009 [10], the present analysis focuses on the association between on-sale alcohol outlet density and violent crime prior to the introduction of off-sale outlets, hence the discontinuation of the dataset in August, 2009. Three archival data sets were employed in the study. First, data pertaining to reports of violent crime (murder, rape, robbery, and aggravated assault) during the time period January, 2005 and August, 2009 were obtained directly from the city of Lubbock Police Department. The dataset contained the date, time, and street address for each violent crime incident. There were a total of 7327 violent crimes for the 56-month time period, of which 49 (0.7%) were murders, 374 (5.1%) rapes, 1422 (19.4%) robberies, and 5482 (74.8%) assaults. Close to 99% of these violent crimes were geocoded by street address using Centrus Desktop. These data were then aggregated to the census block group level and the violent crime rate was calculated as crimes per 1,000 persons.

Second, a list of all alcohol outlets active during the same 56-month time period in the city of Lubbock was obtained from the Texas Alcoholic Beverage Commission (ABC) online database, which includes the name, geographic location, and type of permit or license of the outlet. There were a total of 197 on-sale licenses over the study time period, and outlet density per 1000 residents was used as a measure of alcohol availability. All outlets were geocoded and aggregated to the census block group level in the analysis.

Third, neighborhood population and sociostructural variables were extracted from the 2006–2010 American Community Survey 5-year estimate [11]. Consistent with previous ecological studies of alcohol availability and violence [3, 4], 12 neighborhood sociostructural variables were used as potential covariates in the analysis. These 12 neighborhood sociostructural variables fell into three major categories: (1) *concentrated disadvantage*: % families below poverty, % households receiving public assistance, % unemployed over age 16, % female-headed households with children, % Black, and % Hispanic; (2) *residential instability*: % of residents over age 1 who have lived in the same house 1 year ago, % homes that are owner occupied, % vacant housing units; (3) *sociodemographic measures of the resident population*: adult to child ratio, population density, and % population that is male and aged 15–24.

### 2.2. Statistical Analyses

Multivariate regression analysis was first conducted to examine the relationship between alcohol outlet densities, neighborhood sociostructural characteristics, and violent crime rate. Specifically, a stepwise ordinary least square (OLS) procedure was run to identify significant explanatory variables, with a 0.01 significance level for entry and a 0.05 level for removal. The candidate covariates in the study were the 12 factors pertaining to neighborhood characteristics described previously. Before performing the regression analysis, percent Black and outlet density variables were log transformed to adjust for skew. The dependent variable (violent crime per 1,000 population) was also log transformed, and a small constant was added to transform zero values. Plots of leverage values were used to identify outlying extreme values, and the variance inflation factors (VIF) were obtained for each of the explanatory variables to assess multicollinearity. Residual plots and partial regression plots were also checked for nonrandom pattern and model specification to ensure the inclusion of all the important explanatory variables in the final model. Regressions were also run with mean replacement for a small number of missing neighborhood variables, but results essentially remained unchanged and therefore are not reported.

To explore spatial association in the relationships between alcohol outlet densities, neighborhood sociostructural characteristics and the violent crime rate across the study area, the Geographically Weighted Regression (GWR) method was also employed [12]. The GWR produces a separate parameter estimate of regression coefficients, goodness-of-fit, and significance assessment for each observation. To minimize potential problems associated with multiple testing and multicollinearity among predictor variables, those statistically valid and significant predictors identified using OLS procedures were used in GWR model. For GWR modeling, we used adaptive kernels based on bisquare weighting function due to the irregular shape of the study area [13]. This method is often preferred in identifying the optimal number of nearest neighbors, considering the density and size of samples. To identify the optimal size of kernels, we used the Akaike Information Criteria (AIC) optimization method which identifies the bandwidth that minimizes the AIC score and that accounts for the local variation in the size of the data set [12, 14]. Estimates of spatial autocorrelation (Moran's I) were also obtained to ensure that residuals were not spatially correlated. Monte-Carlo significance tests were conducted to assess statistical significance of nonstationarity for alcohol availability and each of the covariates [15]. The calibration of local GWR models was performed using the GWR 3.0 [12].

## 3. Results

Descriptive summary statistics (mean and standard deviations) for six neighborhood variables included in the model (percent Hispanic, percent Black, percent families below poverty, percent owner occupied, percent residential stability, and population density), on-sale outlet density, and violent crime rates are shown in Table 1. Summary statistics for each individual crime type (assault, robbery, rape, and murder) are also included in the table.

Table 2 presents the summary of regression parameters (coefficients, standard errors, *t* values, and 95% confidence intervals) and diagnostics (adjusted *R*-squared and AIC) for the OLS model of violent crime that was constructed with six neighborhood variables and on-sale alcohol outlet density. These predictors explained about 63% of the variance in violent crime (adjusted *R*-squared = 0.628) and were all statistically significant at *P* values of <0.01. The table shows that a 1% increase in on-sale outlet density was associated with a 0.25% increase in violent crime. Variance inflation factors (VIF) included in the table indicate that multicollinearity among explanatory variables was removed in the model.

**Figure 1:**
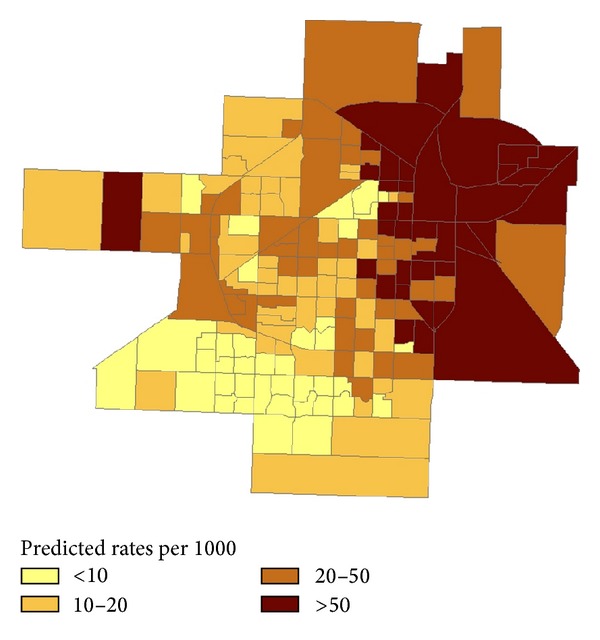
Violent crime rates (per 1000) modeled using Geographical Weighted Regression, Lubbock, Texas, 2005–2009.

The local GWR model was also constructed with the above neighborhood variables and on-sale alcohol outlet density (Table 3 and Figure 1). Figure 1 presents a map of the violent crime rate modeled using the GWR. The map clearly shows spatial patterns of violent crime rates after taking into account local variations of the neighbourhood sociostructural characteristics and including alcohol availability. Specifically, a higher rate of violent crime was identified in the north-east of the city where combined outlet density and neighborhood characteristics have a relatively strong influence. Further, the parameters of each explanatory variable produced by the local GWR model (5-number summary values) differ across the study area with varying degrees of magnitude and sign of the statistical association (Table 3). We also identified block groups with positive and negative values of the *t*-statistic at the 95% level of significance, using standard values of ±1.96. Alcohol outlet density along with several covariates, including percent Black and percent Hispanic, indicated positively significant relationships in a majority of block groups (about 60%), while percent owner occupied and population density showed negatively significant relationships in most block groups within the city (close to 90% of the block groups). Additionally, spatial nonstationarity of predictor variables was further assessed by the *P* values obtained from the Monte-Carlo tests. Two predictor variables (percent Black and percent Hispanic) exhibited statistically significant nonstationarity, while the rest of the variables, including alcohol outlet density, were not statistically significant. We further conducted sensitivity analyses in assessing statistical significance of spatial nonstationarity of predictor variables; results from the Monte-Carlo tests with all 12 sociostructural neighbourhood variables remained unchanged, indicating that errors due to model misspecification unlikely.

Finally, regression coefficients and diagnostic values were used to compare the performance between the two models. As indicated previously, the best OLS model explained slightly less than two-thirds of the variability in violence within the study area, while the GWR improved the model with an additional 7% in explained variance (mean adjusted *R*-squared value of 0.704). The AIC score for the GWR model (361.97) was much smaller than the AIC from the global OLS model (388.46), which suggests that the local GWR model provided a better fit to the data, and thus significant improvement over the global model.

**Figure 2:**
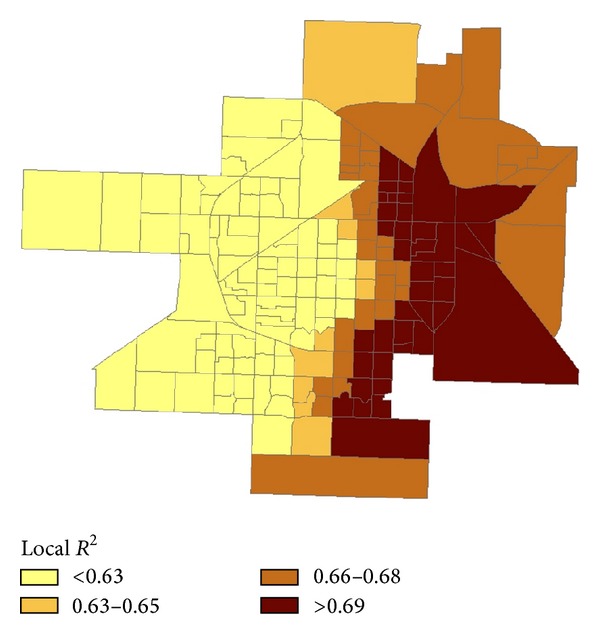
Distribution of local *R*-squared values, Lubbock, Texas, 2005–2009.

The spatial distribution of local *R*-squared values produced by the GWR analysis is also presented in Figure 2. Spatial variations in these local *R*-squared statistics demonstrate how the combined effects of neighborhood population and place characteristics (including outlet density) on violent crime vary across block groups within the city. This map also identifies geographic areas where the GWR produces an improvement in overall model fit with respect to the global model. More than 50% of census block groups showed an improvement over the *R*-squared of 0.63 from the global OLS model, especially in a majority of block groups east of the city.

## 4. Discussion

This study investigated whether violent crime is spatially and/or locally associated with neighborhood sociostructural characteristics, including the availability of alcohol through on-sale premises, in the city of Lubbock, Texas. Using the OLS procedure we identified several neighborhood sociostructural variables, including on-sale alcohol outlet density, that showed a statistically significant association with violent crime in the city, and which explained 63% of the variance in this outcome. An additional 7% was explained by the local GWR model, which explained more than two-thirds of the variability in violence within the study area. We also observed spatially varying population and neighborhood variables associated with violent crime within the study area, often not captured by the global model. The findings provide evidence of the ecological association between on-sale alcohol outlets and violence overall and show that violence is spatially and locally associated with neighborhood and on-sale alcohol environment.

The findings concerning alcohol outlet density and violence add to a growing body of research that has examined the association between these two variables [1, 5]. As noted above, while a positive association between on-sale alcohol outlets and violence has been reported in a number of studies, overall the previous research in this area has produced mixed results with many studies reporting no association [5]. The current study may provide more credible evidence on the inconsistent associations reported previously as it allows assessment of the influence of on-sales outlets on violence in the almost total absence of off-sale outlets. We found that such outlets explained an additional 3% of the variance in violent crime in the OLS model. A number of mechanisms have been proposed as explanations of this relationship.

At the most basic level, alcohol consumption will increase as availability increases and this, in turn, will lead to a rise in both excessive drinking and alcohol-related harms [16]. Beyond availability, it has also been proposed that on-sale outlets can have a deleterious effect on local communities. For example, Livingston and colleagues argue that on-sale alcohol outlets can have an “amenity effect” which operates primarily in terms of the types of individuals that they attract into a neighborhood and the interactions that occur between them following the consumption of alcohol [8]. Along similar lines, Gruenewald's niche theory posits that alcohol outlets market their products to specific segments of the drinking population and that different types of drinkers are attracted to different types of drinking environments some of which are more conducive to the generation of violence than others [9].

The findings presented should be interpreted with caution given the limitations of the study. First, the question as to whether neighborhood characteristics and outlet density are causally linked to the increased violence remains to be answered. Similarly, interpretations of our findings should consider limitations inherent due to the study design and measures used in the study; these include the use of population-based measures of violence and outlet density and potential aggregation problems of outlet density due to the cross-sectional study design. Study designs that take advantages of longitudinal changes in neighborhood characteristics and violence and that employ improved measures of violence (that consider population movement) and outlet density (that consider alcohol sales and duration of outlet operations) may help address this question [17].

Second, we cannot rule out the possibility that there may be other unmeasured neighborhood population and/or place characteristics that may be associated with violence. Other aspects of neighborhood social and built environments that may be directly or indirectly related to violence need to be further investigated, such as social capital, drug availability and other neighborhood institutions [18–20]. These too may require the use of methods other than those employed in our study [21]. Third, the results reported pertain to one isolated city in north-west Texas and may not be generalizable beyond this setting. Lastly, while GWR certainly provides the capability to explore and interpret the significance and sign of spatial and local associations, often undetected by conventional global models, it is not without limitations [22–24]. However, we additionally conducted sensitivity analyses to make sure that important predictor variables were not omitted in assessing statistical significance of spatial nonstationarity and to ensure that potential problems due to multiple testing and multicollinearity were not present by using statistically valid and significant predictors in the model.

## Figures and Tables

**Table 1:** Descriptive summary statistics for violent crime, on-sale alcohol outlets, and neighborhood sociodemographic variables, 2005–2009, Lubbock, Texas (*n* = 170).

	Mean	Standard deviation
Neighborhood variables included in the model		
Percent Hispanic	30.9	24.5
Percent Black	8.6	15.2
Percent families below poverty	16.5	20.0
Percent owner occupied	55.6	27.7
Percent residential stability	73.0	17.7
Population density	4261	2764
Outlet density (per 1000)	1.3	3.9
Violent crime (per 1000)	44.1	75.0
Assault	31.7	42.9
Robbery	10.6	38.1
Rape	2.9	3.2
Murder	1.2	0.9

**Table 2:** Ordinary least squares (OLS) model of violent crime (global model).

Variable	Estimate	Std. error	*t* value	95% CI	VIF^a^
Percent Hispanic	0.021	0.002	8.39	0.016, 0.026	1.169
Percent Black (log)	0.212	0.053	4.01	0.108, 0.317	1.207
Percent families below poverty	0.014	0.003	3.97	0.007, 0.021	1.533
Percent owner occupied	−0.014	0.004	−3.76	−0.021, −0.006	3.097
Percent residential stability	0.014	0.005	2.77	0.004, 0.023	2.384
Population density	−0.0001	0.000	−4.65	−0.000, −0.000	1.208
Outlet density (log)	0.250	0.066	3.78	0.119, 0.381	1.137
Intercept	2.109	0.340	6.19	1.437, 2.781	

Diagnostics					
Adjusted *R* ^2^			0.628		
AICc			388.46		

^a^Variance inflation factors.

**Table 3:** Geographically weighted regression (GWR) model of violent crime (local model).

Variable	Minimum	Lower quartile	Median	Upper quartile	Maximum
Percent Hispanic	0.018	0.021	0.022	0.023	0.024
Percent Black (log)	0.108	0.180	0.233	0.258	0.289
Percent families below poverty	0.001	0.007	0.011	0.015	0.022
Percent owner occupied	−0.025	−0.013	−0.012	−0.011	−0.008
Percent residential stability	−0.004	0.007	0.009	0.013	0.035
Population density	−0.0001	−0.0001	−0.0001	−0.0001	−0.0001
Outlet density (log)	0.071	0.147	0.246	0.346	0.399
Intercept	0.897	2.031	2.304	2.523	3.515

Diagnostics					
Adjusted *R* ^2^			0.704		
AICc			361.97		

## References

[1] Popova S, Giesbrecht N, Bekmuradov D, Patra J (2009). Hours and days of sale and density of alcohol outlets: impacts on alcohol consumption and damage: a systematic review. *Alcohol and Alcoholism*.

[2] Gorman DM, Speer PW, Gruenewald PJ, Labouvie EW (2001). Spatial dynamics of alcohol availability, neighborhood structure and violent crime. *Journal of Studies on Alcohol*.

[3] Zhu L, Gorman DM, Horel S (2006). Hierarchical Bayesian spatial models for alcohol availability, drug “hot spots” and violent crime. *International Journal of Health Geographics*.

[4] Britt H, Carlin BP, Toomey TL, Wagenaar AC (2005). Neighborhood level spatial analysis of the relationship between alcohol outlet density and criminal violence. *Environmental and Ecological Statistics*.

[5] Toomey TL, Erickson DJ, Carlin BP (2012). The association between density of alcohol establishments and violent crime within urban neighbourhoods. *Alcoholism*.

[6] Gruenewald PG, Freisthler B, Remer L, LaScala EA, Treno A (2006). Ecological models of alcohol outlets and violent assaults: crime potentials and geospatial analysis. *Addiction*.

[7] Lipton R, Gruenewald PJ (2002). The spatial dynamics of violence and alcohol outlets. *Journal of Studies on Alcohol*.

[8] Livingston M, Chikritzhs T, Room R (2007). Changing the density of alcohol outlets to reduce alcohol-related problems. *Drug and Alcohol Review*.

[9] Gruenewald PG (2007). The spatial ecology of alcohol problems: Niche theory and assortative drinking. *Addiction*.

[10] Han D, Gorman DM (2013). Evaluating the effects of the introduction of off-sale alcohol outlets on violent crime. *Alcohol and Alcoholism*.

[11] US Census Bureau (2012). *American Community Survey (ACS): 2006–2010 5-Year Estimates*.

[12] Fotheringham AS, Brunsdon C, Charlton ME (2002). *Geographically Weighted Regression: The Analysis of Spatially Varying Relationships*.

[13] Brunsdont C, Fotheringham S, Charlton M (1998). Geographically weighted regression-modelling spatial non-stationarity. *Journal of the Royal Statistical Society Series D*.

[14] Mennis J (2006). Mapping the results of geographically weighted regression. *Cartographic Journal*.

[15] Hope A (1968). A simplified Monte Carlo significance test procedure. *Journal of the Royal Statistical Society Series B*.

[16] Single EW, Chaudron C, Wilkinson D (1998). The availability theory of alcohol-related problems. *Theories on Alcoholism*.

[17] Livingston M (2011). A longitudinal analysis of alcohol outlet density and domestic violence. *Addiction*.

[18] Gorman DM, Zhu L, Horel S (2005). Drug ‘hot-spots’, alcohol availability and violence. *Drug and Alcohol Review*.

[19] Peterson RD, Krivo LJ, Harris MA (2000). Disadvantage and neighborhood violent crime: do local institutions matter?. *Journal of Research in Crime and Delinquency*.

[20] Theall KP, Scribner R, Cohen D, Bluthenthal RN, Schonlau M, Farley TA (2009). Social capital and the neighborhood alcohol environment. *Health and Place*.

[21] Furr-Holden C, Campbell K, Milam A, Smart M, Ialongo N, Leaf P (2010). Metric properties of the neighborhood inventory for environmental typology (NIfETy): an environmental assessment tool for measuring indicators of violence, alcohol, tobacco, and other drug exposures. *Evaluation Review*.

[22] Graif C, Sampson RJ (2009). Spatial heterogeneity in the effects of immigration and diversity on neighborhood homicide rates. *Homicide Studies*.

[23] Páez A, Farber S, Wheeler D (2011). A simulation-based study of geographically weighted regression as a method for investigating spatially varying relationships. *Environment and Planning A*.

[24] Waller LA, Zhu L, Gotway CA, Gorman DM, Gruenewald PJ (2007). Quantifying geographic variations in associations between alcohol distribution and violence: a comparison of geographically weighted regression and spatially varying coefficient models. *Stochastic Environmental Research and Risk Assessment*.

